# Phenotypic variability in a Hungarian patient with the 4q21 microdeletion syndrome

**DOI:** 10.1186/s13039-015-0118-7

**Published:** 2015-03-03

**Authors:** Katalin Komlósi, Balázs Duga, Kinga Hadzsiev, Márta Czakó, György Kosztolányi, András Fogarasi, Béla Melegh

**Affiliations:** Department of Medical Genetics, Clinical Centre, University of Pecs, Szigeti Street 12, Pecs, H-7624 Hungary; Department of Neurology, Bethesda Children’s Hospital, Bethesda Street 3, Budapest, H-1146 Hungary; Szentágothai Research Centre, University of Pecs, Ifjusag Street 20, Pecs, H-7624 Hungary

**Keywords:** Submicroscopic deletion, Array CGH, 4q21, Short stature, Intellectual disability

## Abstract

**Background:**

Interstitial deletions of 4q21 (MIM 613509) have already been reported in more than a dozen patients with deletions ranging from 2 to 15.1 Mb delineating a common phenotype including marked growth restriction, hypotonia, severe developmental delay with absent or delayed speech and distinctive facial features. A minimal critical region of 1.37 Mb accounting for the common features with 5 known genes (*PRKG2, RASGEF1B, HNRNPD, HNRPDL, and ENOPH1*) has been described so far.

**Results:**

Here we report on a 5 year-old Hungarian girl presenting with severe developmental delay, good receptive language but absent spoken speech, short stature, dystrophy, hypotonia, distinctive facies including broad forehead, frontal bossing, downward slanting palpebral fissures, hypertelorism, hypoplastic ear-lobes, anteverted nostrils, short philtrum, small mouth, higharched palate, short, small hands and feet, distally narrowing fingers and clinodactyly. Cerebral MRI showed ventricular dilation and an increase in periventricular signal intensity. After extensive metabolic tests and exclusion of subtelomeric deletions array CGH analysis was performed using the Agilent Human Genome G3 SurePrint 8x60K Microarray (Agilent Technologies, USA), which detected a 4,85 Mb de novo interstitial deletion of 4q21.21-4q21.23. The clinical symptoms only partly overlap with reported 4q21 microdeletion cases. Among multiple annotated genes our patient is also haploinsufficient for the following genes: *RASGEF1B* being a strong candidate for the neurodevelopmental features and *PRKG2* for severe growth delay.

**Conclusion:**

The first Hungarian case of 4q21 deletion adds to the phenotypic spectrum of this novel microdeletion syndrome and underlines the importance of array CGH to uncover the heterogeneous causes of intellectual disability.

## Background

The recent wide-spread use of microarray-based comparative genomic hybridization (array CGH) has extensively aided the elucidation of the underlying cause in patients with severe developmental delay and intellectual disability with dysmorphic features [1]. Interstitial deletions of 4q21 have been reported in about a dozen patients [1-8] with deletions ranging from 2 to 15.1 Mb delineating a common phenotype including marked growth restriction, hypotonia, severe developmental delay with absent or delayed speech, small hands and feet and distinctive facial features as broad forehead, hypertelorism, and prominent central incisors. A minimal critical region of 1.37 Mb accounting for the common features with 5 known genes (*PRKG2, RASGEF1B, HNRNPD, HNRPDL, ENOPH1*) has been described so far [5].

Here, we report the first Hungarian case of 4q21 deletion adding to the phenotypic spectrum of this novel microdeletion syndrome.

## Case presentation

### Results

**Figure 1:**
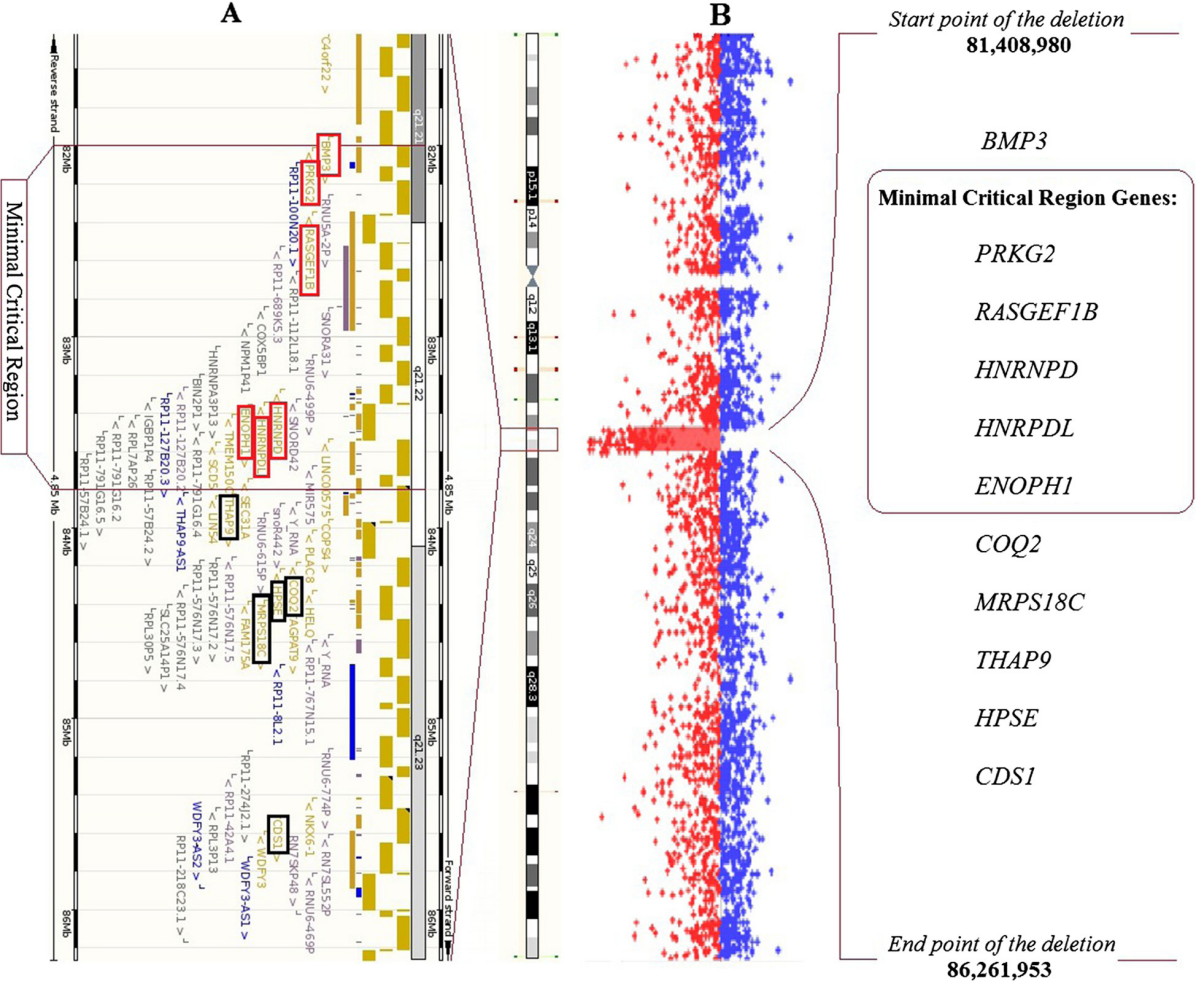
**Ensembl and aCGH image of the 4q21.21-q21.23 deletion.** Part **A** is the Ensembl image of the deleted area with the affected genes and the 4q21 microdeletion syndrome minimal critical region highlighted [9]. Part **B** is our aCGH image where the 4.85 Mb deletion and its exact breakpoints are clearly visible.

After extensive metabolic tests and exclusion of subtelomeric deletions array CGH analysis was performed using the Agilent Human Genome G3 SurePrint 8x60K Microarray, which detected a 4,85 Mb de novo interstitial deletion of 4q21.21-4q21.23 (ch4:81 408 980–86 261 953) (Figure 1). The deletion in our patient involved the following genes: *PRKG2 (MIM 601591), RASGEF1B* (MIM 614532)*, HNRNPD* (MIM 607137)*, HNRPDL, ENOPH1, COQ2, MRPS18C, THAP9, HPSE,* and *CDS1*. Except for known CNVs, no copy number alterations were observed in other chromosomes (data not shown). Based on the normal CGH array profile of the parents this deletion proved to be *de novo*. The deletion was not reported as polymorphic or structural variant in the publicly available databases.

## Discussion

The widespread use of array CGH has led to the delineation of many novel entities associated with developmental delay [1]. Beyond revealing the underlying cause it provides information for prognosis, the medical management of the symptoms and access to resources for the affected families, and also gives the basis for estimating recurrence risks.

Several reports and studies have delineated a 4q21 microdeletion syndrome with a common phenotype including marked growth restriction, hypotonia, severe developmental delay with absent or delayed speech, small hands and feet and distinctive facial features as broad forehead, hypertelorism, and prominent lower and upper incisors [5]. A minimal critical region of 1.37 Mb with 5 known genes (*PRKG2, RASGEF1B, HNRNPD, HNRPDL, ENOPH1*) has been described so far [5]. Comparison of our case with previously published cases (Table 1) revealed several common features but also some variation in the phenotype. The 4.85 Mb deletion in our patient includes the minimal critical region of the 4q21 microdeletion syndrome, encompassing the candidate genes, *PRKG2 and RASGEF1B,* previously described as major determinants of the 4q21 phenotype. Our patient shares haploinsufficiency with the patient portrayed as having the smallest reported deletion in this region, a novel 2.0 Mb deletion encompassing three of the genes in the proposed minimal critical region: *HNRNPD, HNRPDL, and ENOPH1* [7]. The shared features between this patient and our patient, including macrocephaly, small hands and feet, developmental delay, and the distinctive facial features of broad forehead, hypertelorism and prominent lower incisors, stress the role of these genes as likely candidates for the shared phenotype through yet unrevealed mechanisms [7].

**Table 1:** **Phenotypic differences between patients with 4q21 microdeletions and common features of the minimal critical region**

**Clinical/common features**	**Minimal critical region** **[** 5 **]**	**Smallest described deletion** **[** 7 **]**	**Largest described deletion** **[** 5 **]**	**Current case report**
Deletion	4q21.21-21.22	4q21.22-q21.23	4q21.21-q22.3	4q21.21-4q21.23
Size (Mb)	1.37	2.0	15.1	4.85
Age at diagnosis (years)	NA	7	8	5
*Craniofacial features*				
Frontal bossing, broad forehead	Yes	Yes	Yes	Yes
Downslanting palpebral fissures	ND	Yes	ND	Yes
Hypertelorism	Yes	Yes	No	Yes
Anteverted nostrils	ND	ND	Yes	Yes
Short philtrum	ND	ND	Yes	Yes
Hypoplastic ear-lobes	ND	No	Yes	Yes
Small mouth	ND	Yes	ND	Yes
Higharched palate	ND	Yes	ND	Yes
*Developmental delay*	Yes	Moderate	Yes	Yes
Neonatal hypotonia	Yes	No	Yes	Yes
Gross motor delay	Yes	moderate	Yes	Yes
Delayed speech	Yes	Yes	Yes	Yes
Stereotypic movements	ND	ND	ND	Yes
Behavioral disturbance	ND	Yes	ND	Yes
*Anthropometric and skeletal abnormalities*	Yes	Yes	Yes	Yes
IUGR	ND	No	Yes	Yes
Birth weight (centile)	ND	50th	25th	25-50th
Postnatal growth delay	Yes	No	−5SD	−2SD
Conserved head circumference	Yes	+1SD	−0.5SD	+1 SD
Small hands and small feet	Yes	Yes	No	Yes
Brachydactyly	Yes	Yes	No	Yes
*Cerebral imaging abnormality*			Yes	Yes
Ventricular dilation	ND	No	Yes	Yes
Corpus callosum hypoplasia	ND	No	ND	Yes
Cerebellar vermis hypoplasia	ND	No	ND	No
Frontal cerebral hypoplasia	ND	No	Yes	No

Yes: feature present; no: feature absent; ND: data not accessible, NA: not applicable.

Among the dysmorphic features the characteristic brachydactyly observed in other patients was less pronounced in our index patient, and no shorter 2nd toe was observed as described in the classic phenotype. Joint laxity and hypermobile joints, also observed in our patient, have been described recently in a patient with a proximal 4q interstitial deletion of 24.89 Mb encompassing 4q12–4q21.21 [10] and is only known in 3 additional patients with proximal 4q deletions. In addition to the common neurocognitive characteristics seen in most 4q21 cases our patient also exhibited stereotypic movements and a behavioral disturbance including occasional self-injurious behavior and aggression towards others as described in the patient with the 2 Mb deletion [7]. It is also noteworthy that good receptive language and communication by sign was observed in our patient besides absent speech also resembling the described case with a large proximal 4q deletion [10], however, more studies with the precise description of the deletion boundaries will be needed to point out genes responsible for the overlapping features.

The 4,85 Mb region involved in the deletion contains a number of genes, some of which have already been discussed as being major determinants of the phenotype [1,5], while the role of other genes and their impact on the phenotype still need to be elucidated. We have learned from previous works that haploinsufficiency of the minimal critical region is essential for the expression of the classic 4q21 phenotype, within this region, the genes *PRKG2 and RASGEF1B*, have been identified as major determinants in the development of the characteristic features [5]. *RASGEF1B* encodes a highly conserved guanine nucleotide exchange factor for Ras family proteins. This protein superfamily is involved in various basic cellular functions such as signal transduction, cytoskeleton dynamics and intracellular trafficking. It is highly expressed in the central nervous system, and may play a role in actin and microtubule dynamics regulating both dendrite and spine structural plasticity [11]. Since several genes related to intellectual disability have been identified in the Rho-GTPase signaling pathway, *RASGEF1B* seems to play a role in the cognitive features of the 4q21 phenotype [5].

There is strong evidence that the second basic feature of the microdeletion syndrome, severe growth delay, can be attributed to the *PRKG2* gene which encodes a cGMP-dependent protein kinase type II protein. Mice with a null mutation of this gene developed postnatal dwarfism as a result of severe endochondral ossification defect at the growth plates and impaired chondrocyte growth. While small hands, short fingers and feet were described, no postnatal growth delay was observed in the patient with the smallest described deletion [7] not containing the *PRKG2* gene, while in patients deleted for the minimal critical region and in our current case growth delay was severe (Table 1). Those observations also underline that the haploinsufficiency of the *PRKG2* gene could explain growth failure [7] in most of the 4q21 patients. On the other hand, haploinsufficiency of *PRKG2* has previously also been linked to severe cognitive developmental delay [12].

Additionally the deletion in our patient also involved several genes (*BMP3, COQ2, MRPS18C, THAP9, HPSE, and CDS1*) for which no direct function can be linked to the features observed in our patient. In rat embryos, Bmp3 was suggested to be involved in pattern formation during early skeletal development [13]. In Bmp3 −/− embryos or newborns no skeletal defects were found, only increased trabecular metaphyseal bone density and total trabecular bone volume [14]. On the other hand, a missense mutation in the *BMP3* gene (F452L) was associated with cranioskeletal differences in canines [15]. Strehle et al. argued that haploinsufficiency of *BMP3* might be associated with short stature and other skeletal anomalies in 4q21 interstitial deletions [16]. Thus, it cannot be ruled out that haploinsufficiency of *BMP3* may also contribute to the cranial features observed in our patient, such as broad forehead and frontal bossing.

The *HPSE* gene encodes a heparanase belonging to the family of endoglycosidases which cleave the heparan sulfate side chain of heparan sulfate proteoglycans (HSPGs) and contribute to the remodeling of the extracellular matrix for cell movement or the release of bioactive molecules from the extracellular matrix or cell surface [17]. Vlodavsky et al. demonstrated a correlation of *HPSE* expression and heparanase activity with increased metastatic potential in breast cancer tissues and cell lines [18]. Currently no direct function of the *HPSE* gene can be linked to developmental disorders, however, it can be assumed that the basic function of extracellular matrix remodeling might also be essential for neurodevelopment. Further detailed case reports or experimental data are needed to learn more about the clinical relevance of those genes.

## Conclusions

We describe the first Hungarian patient with a de novo previously unreported interstitial 4q deletion, syndromic severe developmental delay, absence of spoken language and behavioral disturbance. The clinical symptoms in our patient partially overlap with reported 4q21 microdeletion cases. Among the multiple annotated genes our patient is also haploinsufficient for *RASGEF1B,* a strong candidate for the neurodevelopmental features and *PRKG2* for severe growth delay. In the future elucidation of the clinical relevance of several other deleted genes in 4q21 patients may help establish guidelines for adequate healthcare management of those patients. Our case of 4q21 deletion adds to the phenotypic spectrum of this novel microdeletion syndrome and underlines the importance of array CGH to uncover the heterogeneous causes of intellectual disability.

## Methods

### Patient report

The patient was a 5 year old girl born by caesarean section at 39th week of gestation as the second child of non-consanguineous healthy Hungarian parents, the family history was unremarkable. Her birth weight was 2750 g (25–50 pc), her length 49 cm (5–10 pc), the head circumference 36 cm (+1SD). Her 5 and 10 minute Apgar scores were 9/10. In the perinatal period mild icterus, joint laxity in the hips, axial hypotonia and poor feeding was noted. At 1 week of age severe axial hypotonia and spasticity in the lower limbs was recognized and there was only slight improvement following extensive neurohabilitation. After 3 months her somatic and psychomotor development slowed down and has been very slow ever since. At 6 months of age the patient was hospitalized with severe obstructive bronchitis and during her first year she suffered several upper airway infections with dense mucous and chronic diarrhea, but *CFTR*-related diseases were excluded. At 14 months of age brain MRI revealed significantly widened and abnormally structured ventricles, diminished periventricular white matter and hypoplasia of the corpus callosum. At the age of 18 month the patient was referred to our genetic counseling unit because of severe hypotonia and developmental delay. Postnatal growth delay: weight was 9.5 kg (5–10 pc), height 68 cm (<3 pc) and head circumference 48.5 cm (+1 SD) and a distinctive facies including broad forehead, frontal bossing, downward slanting palpebral fissures, hypertelorism, hypoplastic ear-lobes, anteverted nostrils, short philtrum, small mouth, higharched palate as well as short, small hands and feet, distally narrowing fingers, clinodactyly and joint laxity were noted. Neurological examination revealed severe generalized hypotonia and absent speech development. Gross motor milestones were severely delayed despite of extensive neurohabilitation: at the age of 2.5 years she was unable to sit alone, she did not crawl and was unable to stand alone. At the age of 5 years she was able to walk, sit alone, but had no speech. She had good receptive language and used signs and gestures to communicate but had no speech. Stereotypical movements such as hand clapping and flapping and a behavioral disturbance, including occasional self-injurious behavior and aggression toward others were observed. Epilepsy has not been noted so far and repeated EEGs gave negative results. Extensive metabolic (carnitine-ester profiling, amino acids, urine organic acids, isoelectric focusing for CDGs) and genetic testing (routine karyotyping, *CFTR* sequencing, mitochondrial mutation screening) yielded negative results.

### Array Comparative Genomic Hybridization (aCGH) analysis

Array CGH was performed using the Agilent Human Genome G3 SurePrint 8x60K Microarray (Agilent Technologies, USA), a high resolution 60-mer oligonucleotide based microarray containing 55.077 60-mer probes, spanning coding and non-coding genomic sequences with median spacing of 33 kb and 41 kb, respectively.

Purification of the DNA from blood was performed using the DNA Purification Kit NucleoSpin®Dx Blood (Macherey-Nagel, Germany) according to the manufacturer’s protocol. Concentration and purity of the extracted DNA were measured with the NanoDrop spectrophotometer (NanoDrop Technologies, Inc.). Pooled genomic DNA from peripheral blood leukocytes of phenotypically normal males or females from Promega was used as a reference (Promega Male/Female Reference DNA, Promega Corporation, USA).

Labeling and hybridization were carried out based on the Agilent protocol (Agilent Oligonucleotide Array-Based CGH for Genomic DNA Analysis – Enzymatic Labeling Protocol v7.2; July 2012). Array image was acquired using an Agilent laser scanner G2565CA (Agilent Technologies, California, USA) and analyzed with the Agilent Feature Extraction software (v10.10.1.1.). Results were presented by Agilent Cytogenomics software (v2.5.8.11). DNA sequence information refers to the public UCSC database (Human Genome Browser, Feb 2009 Assembly; GRCh37:hg19).

The deletion detected was aligned to known aberrations listed in publicly available databases, such as the DECIPHER (Database of Chromosomal Imbalance and Phenotype in Humans using Ensembl Resources) [19], DGV (the Database of Genomic Variants) [20], Ensembl [21] and ECARUCA [22]. Parental samples were analyzed using the same array and method.

## Consent

Written informed consent was obtained from the patient for publication of this Case report and any accompanying images. A copy of the written consent is available for review by the Editor-in-Chief of this journal.

## Abbreviations

MRI: Magnetic Resonance Image
a(rray) CGH: Array Comparative Genomic Hybridization
EEG: Electroencephalography
CDG: Congenital Disorder of Glycosylation
CFTR: Cystic fibrosis transmembrane conductance regulator
GRCh37:hg19: Genome Reference Consortium human 37/human genome 19
UCSC database: University of California, Santa Cruz Genome Browser database
DECIPHER: Database of Chromosomal Imbalance and Phenotype in Humans using Ensembl Resources
DGV: The Database of Genomic Variants
ECARUCA: European Cytogeneticists Association Register of Unbalanced Chromosome Aberrations
CNV: Copy Number Variation
